# Mental Health of Healthcare Workers During the COVID-19 Pandemic

**DOI:** 10.14789/jmj.JMJ21-0032-R

**Published:** 2022-02-04

**Authors:** NARIMASA KATSUTA

**Affiliations:** 1 Department of Psychiatry, Juntendo University Faculty of Medicine, Tokyo, Japan; 1 Department of Psychiatry, Juntendo University Faculty of Medicine, Tokyo, Japan

**Keywords:** COVID-19, healthcare workers, mental health, pandemic, Japan

## Abstract

On January 16, 2020, the first case of coronavirus disease 2019 (COVID-19) in Japan was reported. In the spring of the same year, the shortage of personal protective equipment (PPE) such as surgical masks became a significant issue. In addition, the medical staff had to encounter discrimination during this period. Thus, the mental health of these staff has been greatly affected by the social situation, the media coverage of the COVID-19 epidemic, and the shortage of PPE in hospitals.

Various factors make it difficult for the medical staff to seek professional help for mental well-being. Therefore, self-care plays an important role in the prevention of depression and anxiety disorders among healthcare workers. When the healthcare workers face problems in their work environment, they should coordinate with the hospital to promptly improve the system. COVID-19 resulted in new societal norms and changed our lifestyles significantly. Insomnia is a particular issue among healthcare workers. Lifestyle analysis is thus necessary if insomnia needs to be addressed.

Because the opportunities for communication are reduced during the COVID-19 pandemic, conscious communication is essential. During this difficult time, the staff may not receive sufficient guidance from their superiors at work, for example for guidance received by resident doctors from their seniors. This will also provide opportunities to communicate vital information about matters such as infection control. Therefore, quality communication and accurate information should be directed toward all healthcare workers.

## Introduction

In the winter of 2019, a new viral pneumonia was detected in Wuhan, China, and the causative pathogen was identified as severe acute respiratory syndrome coronavirus-2 (SARS-CoV-2). On January 16, 2020, the first case of coronavirus disease 2019 (COVID-19) in Japan was reported. Since then, COVID-19 has spread across the globe. In the spring of the same year, patients began to complain of suspected COVID-19 at their local medical practices based on misinformation about the growing epidemic. Healthcare workers were inundated with demands for tests and treatment. In addition, the shortage of personal protective equipment (PPE) such as surgical masks became a big problem during this period. Many medical institutions had to impose restrictions such as one mask per person per week. The supply of gowns ran out, and aprons fashioned from plastic bags had to be used instead. At that time, there was no cure for the new coronavirus, and infection control guidelines had to be hastily established. Both the “sword” of treatment and the “shield” of PPE proved inadequate against COVID-19.

In addition, there was discrimination against medical staff during this period. Specifically, medical professionals who responded to COVID-19 were bullied in the workplace and discriminated against by children’s nursery schools and kindergartens, which prohibited these healthcare workers from going to kindergartens.^1)^ Children of healthcare workers were bullied and harassed, denied admission to nursery schools, and refused carriage by taxi drivers.^2)^ A study of 10,511 healthcare workers who responded to SARS found that 49% were subject to social criticism and 31% to family inhibition. The damage to mental health among these workers caused by discrimination is a matter that requires attention.^3)^

The mental health of medical staff has been greatly affected by the social situation, the media coverage of the COVID-19 epidemic, and the PPE shortages in hospitals. When considering the mental health of medical professionals, it is necessary to address and resolve the problems with society and the work environment.

## Self-care of healthcare worker

Various factors can make it difficult to ask for professional help with mental health problems. These include stigma, inactivity, cost, convenience, and the belief that the problem can be resolved alone.^4)^ Therefore, the prevention of depression and anxiety disorders among healthcare workers requires individual self-care.

The first step in self-care is awareness. The COVID-19 pandemic might be comparable to the Black Death of the 14th century, the cholera outbreak of the 19th and 20th centuries, and the Spanish flu of 1918. The impact this global health crisis has had on mental health is immeasurable. The hard work of healthcare workers across the world deserves a great deal of praise. They are at the forefront of the fight against a global crisis the likes of which are seen once in hundreds of years. It is necessary to understand that worry is a natural response to such a difficult situation. Self-care must include appropriate self-analysis and concrete measures to solve the emotional and practical problems one faces. Work satisfaction is negatively correlated with psychosocial issues so dealing effectively with issues at work is vital to mental health.^5)^ WHO provides useful guidelines on specific methods of self-care.^6)^ Interested readers should consult the web link in the reference for further details.

When medical staff encounter problems in the work environment, it is important to coordinate with the hospital to promptly improve the system. This paper will consider the efforts made to address the possible detrimental effects of COVID-19 on mental health among healthcare workers at the Juntendo Clinic of the Juntendo University School of Medicine. These efforts have focused on encouraging self-care among staff. Specifically, this has consisted of self-care through improvements to lifestyle and improved communication. We shall consider each of these in turn.

## Improvements in lifestyle

COVID-19 created new societal norms and changed our lifestyles significantly. Insomnia is a particular issue among healthcare workers. There have been numerous reports of insomnia among healthcare workers dealing with COVID-19.^7)^ Causes of insomnia can include stress, lack of exercise, drinking and smoking, evening caffeine intake, and browsing smartphones and computers before going to bed. Because COVID-19 restricts social activity, individuals find themselves at home more and this can lead to lethargy and inactivity. Lifestyle analysis is necessary if insomnia is to be addressed. Does the individual skip meals or overeat? Do they drink a lot of alcohol alone at home? Are they absorbed in social media until just before going to bed? Such bad habits should be corrected immediately.

To improve quality of life, one must make the conscious decision to “Act-Belong-Commit.”^8)^ “Act” means engagement in activities one enjoys. It may include taking a walk, listening to music, and talking to or spending time with friends. “Belong” refers to engagement with other people; this means belonging to social groups and actively participating in events or hobby groups. “Commit” refers to involvement in, and commitment to, activities, causes, or organizations. Mental health can be maintained by actively engaging in desirable activities to achieve positive life changes.

Excessive exposure to information about COVID- 19 during off-hours is not desirable. A great deal of the information about COVID-19 on the Internet is based on insufficient evidence and is often purposely emotive. It is important to switch off one’s mind before going to bed to prevent insomnia. It is also necessary to consciously select the information to which we attend. Healthcare workers dealing with COVID-19 during working hours should take particular care to avoid too much attention to the topic outside of work to ensure a good balance between work and private life. Individuals exposed to disasters often read and watch more media coverage of the event than others.^9)^ This is probably an attempt to better understand the events they have been affected by but it can be detrimental to mental health to overexpose oneself to such negative information. A Chinese study of the COVID-19 pandemic found that more exposure to COVID-19 social media coverage was associated with more adverse effects, anxiety, and depression among adults.^10)^ To create a clear distinction between work and private life, healthcare workers should avoid COVID-19 news at home and immerse themselves instead in enjoyable activities and hobbies.

## Improvements in communication

Since opportunities for communication are reduced during the COVID-19 pandemic, it is necessary to communicate consciously. If stress accumulates and there is no opportunity to discuss work worries and complaints, it can be difficult to maintain a healthy mentality. During this difficult time, there may be insufficient guidance available from work superiors, for example for residents from senior doctors. It is advisable to try to create opportunities for regular communication between higher and lower-ranking members of staff, including new employees. This will also provide opportunities to communicate vital information about matters such as infection control.

It is important to provide all staff with quality communication and accurate information updates. Explanations should be clear, honest, and frank, and every effort should be made to implement procedures that help everyone to work safely and comfortably. It also provides staff with opportunities to discuss their experiences, provide mutual support, and increase social cohesion.^11)^ Smooth communication should be optimized not only between senior and junior staff but also between colleagues.

Employees who live alone may feel emotionally isolated and it is important to encourage these workers to maintain regular connections with family and friends. In a cross-sectional study in China, medical staff living alone during the COVID-19 pandemic reported significantly higher depressive symptoms than those living with others.^12)^ A study of Japanese medical institutions also found that the depressive symptoms of healthcare workers tended to decrease as the number of cohabitants increased.^13)^ For this reason, daily communication with others is important to the prevention of depression.

## Support for healthcare workers at Juntendo University Hospital

At Juntendo University Hospital, we have secured adequate supplies of vital COVID-19 medical items such as PPE. It is important to stockpile supplies for potential emergency situations in advance and we must not spare money to protect our staff. Staff care issues should not be underestimated, and it is desirable to take prompt action when problems occur. Staff care issues include salary, working hours, vacations, and breaks. Under busy circumstances and in crisis situations such as COVID-19, it is desirable to avoid salary cuts. In addition, since the COVID-19 pandemic is expected to be an issue in healthcare for some time, it is important to reduce overtime as much as possible and create a workplace atmosphere that encourages all employees to take vacations.

The health management office of Juntendo University Hospital has provided counseling and mental health support for healthcare workers in high-stress departments. Employees who develop depression often lack the motivation or drive to seek the help they need on their own. When staff develop mental health issues and take a leave of absence, it can take time to recover, so it is in the interests of the organization as well as its employees for the employer to take the initiative in preventing depression. The mental health department of Juntendo University Hospital has enhanced the available support system in response to the pandemic to ensure it can always respond to staff issues related to COVID-19. Psychiatric consultation is recommended because it is often necessary to prescribe psychotropic drugs for employees who have marked insomnia and apparent deterioration in daytime performance, or are suffering from depressed mood or anxiety.

## The results of the 2021 mental health check at our hospital

This observational cohort study was conducted in June 2021 as part of a mandatory health check of Juntendo University Hospital employees (Tokyo, Japan). A total of 4,350 participants completed a web-based questionnaire on their medical history and current health status. The Center for Epidemiologic Studies Depression Scale (CES-D) was used for assessment, with a score of ≥16 considered indicative of depression. The study protocol was approved by the Ethics Committee of the Juntendo University Faculty of Medicine (approval no. 22004). Informed consent was obtained from all participants. Statistical analyses were performed using SPSS v. 22 (IBM Corp., Armonk, NY, USA). Chi-square tests were performed to assess correlations between depression scores and patient characteristics (e.g., sex). Clinical variables were compared using two-tailed Mann-Whitney U tests in cases with two groups or the Kruskal-Wallis test in cases with three or more groups. A two-tailed *p*-value of <0.05 was considered significant for all tests.

Correlations between variables associated with the CES-D scores were subjected to univariate analyses.

In this study, the prevalence of depression among all employees was 30.8% in 2021, and significantly greater than the pre-pandemic value in 2019 of 27.5%. When participants were subdivided by occupation (nurses, paramedics, doctors, residents, clerks, researchers, support staff, teaching staff, and part-time staff), nurses had the highest depression rate (41.5%), followed by clerks (33.4%), support staff (32.7%), paramedics (31.9%) , and researchers (27.2%), whereas teaching staff (21.8%), residents (20.7%), doctors (20.2%), and part-time staff (17.1%) reported lower depression rates (Table 1). CES-D scores were significantly positively correlated with age (*p* < 0.0001) and sex (*p* < 0.0001). In addition, occupation was associated with CES-D score changes between 2019 and 2021 (*p* = 0.001). The results from binary logistic regression analyses for the score changes between 2019 and 2021 showed that female staff, younger employees, and nurses have a higher risk of depression among Japanese health workers (Table 2). This result is similar to that of previous studies overseas. Luo identified female sex, nursing, and financial poverty as risk factors for anxiety and depression among healthcare workers during COVID-19.^14)^ Xiong also found associations between psychological stress and being female, being under 40, having existing chronic mental illness, and being unemployed in a systematic review.^15)^ The hypothalamic–pituitary–adrenal (HPA) axis was found to be sex-specific; therefore, stress responses differ between men and women.^16)^ Neurobiological and immunological sex differences are also noted, and women are about twice as likely to be depressed as men.^17)^

**Table 1:** Comparison of 2021 and 2019 CES-D results for Juntendo University Hospital employees (Tokyo, Japan).

	Outcome of CES-D		
	2021	2019	
	N = 4350	N = 4240	*p*-value
Sex, M/F	1603/2747	1633/2607	0.114
Age, years	37.8 ± 12.1	37.8 ± 12.1	0.812
Number and the positive rate of CES-D by occupation			
doctor	604(20.2%)	611 (18.0%)	0.343
resident	87 (20.7%)	98 (19.4%)	0.856
nurse	1329 (41.5%)	1295 (40.0%)	0.427
paramedics	499 (31.9%)	501 (30.5%)	0.682
support staff	110 (32.7%)	100 (30.0%)	0.766
clerk	467 (33.4%)	397 (28.7%)	0.142
teaching staff	280 (21.8%)	241 (14.9%)	0.055
researcher	688 (27.2%)	648 (20.5%)	**0.005**
part-time	286 (17.1%)	349 (15.2%)	0.516
Average score by occupation			
doctor	9.0 (4.0 – 14.0)	6.0 (2.0 – 12.0)	**<0.001**
resident	10.0 (6.0 – 14.0)	6.5 (2.0 – 12.25)	**0.005**
nurse	13.0 (8.0 – 21.0)	13.0 (6.0 – 20.0)	**0.002**
paramedics	12.0 (7.0 – 18.0)	10.0 (5.0 – 17.0)	**0.002**
support staff	13.0 (8.0 – 18.0)	10.0 (5.25 – 7.75)	0.175
clerk	11.0 (6.0 – 18.0)	9.0 (4.0 – 16.5)	**0.008**
teaching staff	8.0 (3.0 – 14.0)	5.0 (2.0 – 11.0)	**<0.001**
researcher	10.0 (6.0 – 16.0)	8.0 (3.0 – 13.75)	**<0.001**
part-time	7.0 (3.0 – 12.0)	5.0 (2.0 – 11.0)	**0.006**

*p* values with statistical significance are in bold. The Center for Epidemiologic Studies Depression Scale, CES-D.

**Table 2:** Factors related to CES-D results as determined by logistic regression analysis.

	Odds ratio	95% CI	p-value
Sex			
female	1.18	(1.05–1.32)	**0.007**
male	1		
Age, years	0.98	(0.98–0.99)	**<0.001**
Occupation			
doctor	1.21	(0.93–1.56)	0.156
resident	0.97	(0.63–1.48)	0.882
nurse	2.59	(2.03–3.30)	**<0.001**
paramedics	1.98	(1.54–2.56)	**<0.001**
support staff	2.32	(1.62–3.33)	**<0.001**
clerk	2.12	(1.64–2.75)	**<0.001**
teaching staff	1.31	(0.96–1.78)	0.086
researcher	1.38	(1.07–1.77)	**0.012**
part-time	1		

*p* values with statistical significance are in bold. The Center for Epidemiologic Studies Depression Scale, CES-D.

Nurses were at the forefront of COVID-19 practice and had high infection and mortality rates.^18-19)^ Approximately 10.1% of people infected with COVID-19 are health care workers, and guidance on infection control and mental care for nurses is necessary.^20)^ Nurses, who are mostly women, may have been more prone to depression as they work in the close proximity of patients with COVID-19. Therefore, adequate mental health care may be particularly vital for young female nurses.

## Conclusions

The COVID-19 pandemic has put all healthcare workers under a great deal of psychological stress. To prevent depression in this population, it is important to raise awareness of self-care practices that improve lifestyle habits and increase communication. Younger generations of workers and specific healthcare occupations such as nurses are more vulnerable to depression at this time and require more support.

## Funding

This study was funded by the Juntendo Mental Health Institute (2020-003).

## Author contributions

NK contributed to the conceptualization, design, and writing of this manuscript.

## Conflicts of interest statement

The author declares no conflicts of interest.
